# Study smart: Peer-teaching workshop series for learning strategies, time and stress management in medical studies – a project report

**DOI:** 10.3205/zma001744

**Published:** 2025-04-15

**Authors:** Georg Gross, Leon Feron, Felix Beetz, Nicolas Krapp, Katrin Schüttpelz-Brauns

**Affiliations:** 1 Medical Faculty Mannheim, Heidelberg University, Fachschaft Medizin Mannheim, Mannheim, Germany; 2 Charité – Berlin University of Medicine, Berlin, Germany; 3 Augsburg University, Department of Medical Education Augsburg DEMEDA, Augsburg, Germany; 4 University Medical Centre Augsburg, Internal Medicine III – Gastroenterology, Augsburg, Germany; 5 Medical Faculty Mannheim Heidelberg University, Division for Study and Teaching Development, Medical Education Research Department, Mannheim, Germany

**Keywords:** medical education, peer teaching, self-regulated learning, mental health, resilience

## Abstract

**Introduction:**

Studying medicine places high demands on students' learning behavior. Nevertheless, most medical faculties in German-speaking countries do not offer curricular training for the acquisition of key learning skills. The aim of the project was to develop a series of workshops on evidence-based learning strategies, time and stress management.

**Project description:**

The “study smart” workshop series was developed by students of the Mannheim Medical Faculty in 2018. Participants learn evidence-based techniques for long-term knowledge acquisition, self-, time- and stress management. Due to high demand, the workshop was subsequently incorporated as a permanent component of the curriculum. Since 2021, “study smart” has been a project of the German Medical Students’ Association. Once yearly, students from other faculties are trained at a peer-training-weekend. In total, the workshop series was offered at 8 faculties and several times online. In 2021, a one-off survey of medical students was also conducted at the Medical Faculty Mannheim two months after the workshop series.

**Results:**

A total of 68 workshop participants and 72 non-participants took part in the survey. This showed a more frequent use of the “active recall” learning strategy, in particular the “anki” flashcard program (14 (33%) of participants vs. 4 (10%) of non-participants; p=0.009). Participants reported creating Anki decks, which are shared with the entire class and revised together. In total, over 1000 medical students across Germany have participated in the workshop series over the past 5 years.

**Discussion:**

Student interest in the workshop series remains high. Study Smart has become a widely utilized and highly regarded program. In the assessment of satisfaction with their own learning strategies, there was no significant difference between participants and non-participants. There are also challenges in the long-term implementation of the workshops at medical faculties at other universities due to a high turnover of peer teachers. The national project coordinator is therefore working on establishing local group networks.

## Zusammenfassung

**Einleitung:**

Das Medizinstudium stellt hohe Anforderungen an das Lernverhalten der Studierenden. Dennoch gibt es an den meisten Medizinfakultäten im deutschsprachigen Raum keine curricularen Angebote zum Erwerb von Lern-Schlüsselkompetenzen. Ziel des Projekts war die Entwicklung einer Workshopreihe zu evidenzbasierten Lernstrategien, Selbst-, Zeit- und Stressmanagement.

**Projektbeschreibung:**

In der von Studierenden der Medizinischen Fakultät Mannheim 2018 entwickelten Workshopreihe „Study Smart“ erlernen die Teilnehmenden evidenzbasierte Techniken zum langfristigen Wissenserwerb, Selbst-, Zeit- und Stressmanagement. Aufgrund der großen Nachfrage wurde das Projekt in der Folge curricular verankert. Seit 2021 ist „Study Smart“ ein Projekt der Bundesvertretung der Medizinstudierenden in Deutschland. Einmal jährlich werden Studierende anderer Fakultäten auf einem Training-Peer-Trainer-Wochenende ausgebildet. Insgesamt wurde die Workshopreihe an 8 Fakultäten sowie mehrfach online angeboten. 2021 wurde außerdem an der Medizinischen Fakultät Mannheim eine zwei Monate nach Workshopreihe durchgeführte, einmalige Befragung der Medizinstudierenden durchgeführt.

**Ergebnisse:**

An der Befragung nahmen insgesamt 68 Workshop-Teilnehmende und 72 Nicht-Teilnehmende teil. Hier zeigte sich eine häufigere Nutzung der Lernstrategie „Active Recall“, insbesondere des Karteikartenprogramms „Anki“ (14 (33%) der Teilnehmenden vs 4 (10%) der Nicht-Teilnehmenden; p=0,009). Teilnehmende berichteten von der Erstellung von Anki-Decks, welche mit dem gesamten Jahrgang geteilt und gemeinsam überarbeitet werden. Insgesamt haben deutschlandweit in den vergangenen 5 Jahren über 1000 Medizinstudierende an der Workshopreihe teilgenommen.

**Diskussion:**

Das studentische Interesse an der Workshopreihe ist ungebrochen hoch. „Study Smart“ hat sich als viel genutztes Angebot etabliert. In der Bewertung der Zufriedenheit mit den eigenen Lernstrategien zeigte sich kein signifikanter Unterschied zwischen Teilnehmenden und Nicht-Teilnehmenden. Weiterhin zeigen sich Herausforderungen in der dauerhaften Implementierung der Workshops an Medizinfakultäten anderer Hochschulen aufgrund hoher Fluktuation der Peer-Teacher. Hierfür arbeitet die nationale Projektkoordination an einer Etablierung von Lokalgruppen-Netzwerken.

## 1. Introduction

**Figure 1:**
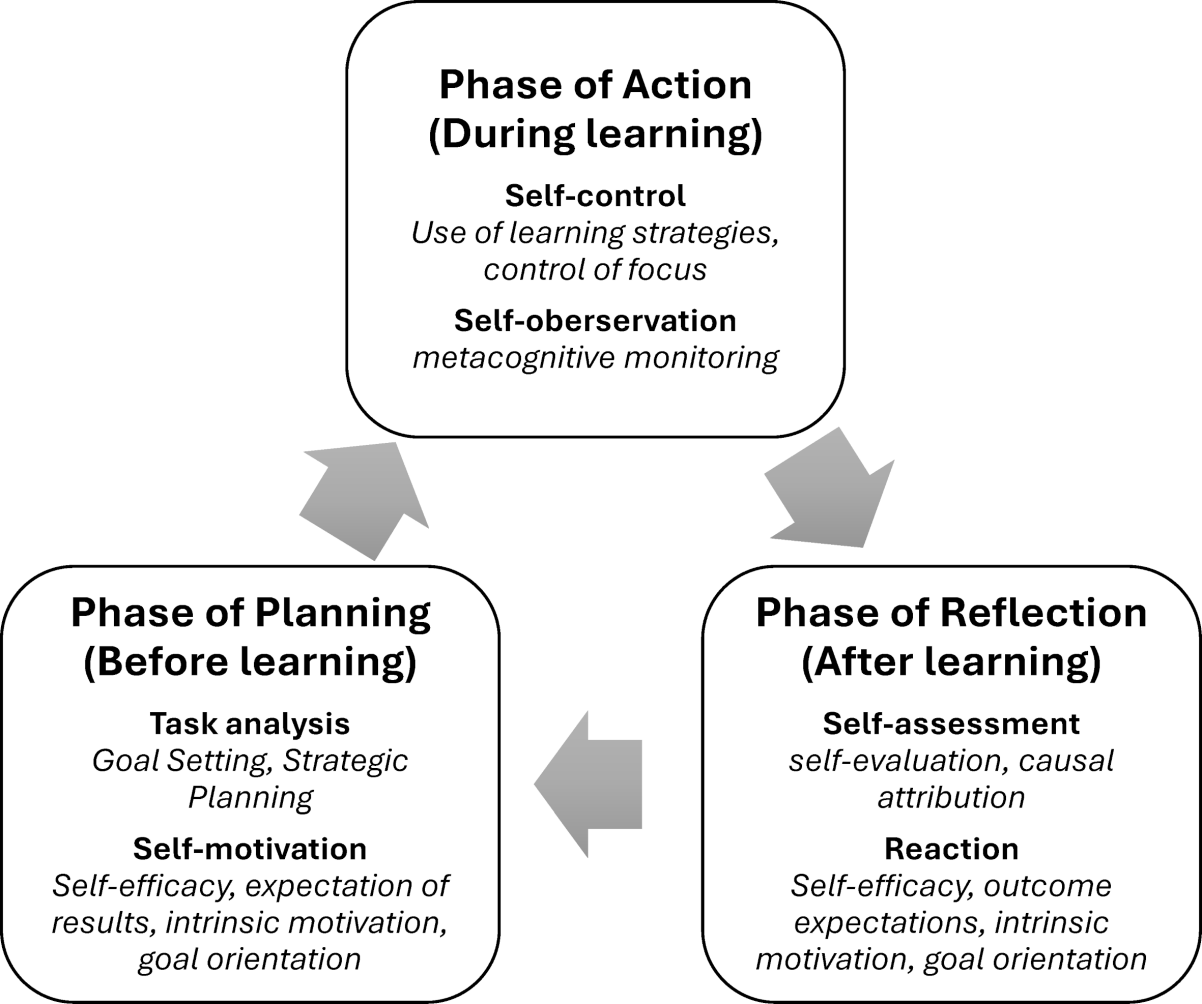
Phases of SRL, adapted from [3] and [24]

Studying medicine places significant demands on students’ learning behavior. From the very first semester, they face examinations that, compared to their university entrance qualification, cover a much greater volume of material, require new skills, and necessitate the adoption of novel learning strategies. At the same time, many students experience considerable subjective pressure to perform. To master the challenges of studying, students need to acquire self-regulation skills [1], [2], [3]. In self-regulated learning (SRL), learning is carried out, monitored and evaluated by the learner in a self-determined manner (see figure 1 (Fig. 1)).

The prerequisite for this is a high level of self-efficacy, i.e. the conviction that you have or can acquire the necessary skills to achieve certain results [4]. 

These skills include key learning competencies such as the use of effective learning techniques or time and stress management. However, studies have shown deficits in these skills among medical students [5], [6]. This is also reflected in our local experience.

Effective learning techniques are characterized by an interconnected understanding of the material (“mastery”), which promotes the long-term recall of knowledge [7]. On the other hand, there is superficial learning, which is characterized by a false sense of familiarity with the content (“fluency”) while at the same time quickly forgetting what has been learned [7]. The most commonly used learning techniques in the first semester are, for example, “writing summaries” or “underlining what has been learned”, but these techniques are mainly used to promote fluency [8]. In a meta-analysis, Hattie et al. examined 51 interventions to promote learning strategies and found a moderate effect on average [9]. Therefore teaching of learning strategies is possible in principle.

In addition, medical students have a significantly higher rate of stress-related illnesses such as depression, burnout and substance abuse compared to the general population [10]. Burger et al. showed that students do not have an increased rate of stress-related illnesses at the beginning of their studies, but that these increase by 13.5% within the first two years of study, thus more than double [11]. There is a connection between self-efficacy (i.e. the conviction that you can master a situation with your own resources) and resilience to stress-related illnesses [12]. It therefore seems coherent to make both effective learning strategies and strategies for dealing with stress available as curricular content early on in the course.

This formed the basis for our project’s aim: to develop a needs-based workshop series to teach students evidence-based learning strategies, self-management, time management, and stress management. The workshops were designed to be accessible and transferable to other medical faculties. This project report outlines the ongoing development of this workshop series.

## 2. Project description

The development, implementation, evaluation and iterative adaptation of the workshop series was carried out using the core cycle [13]. 

### 2.1. Problem identification/general needs assessment

To our knowledge at the time of writing this project report (04/2024), courses to promote key learning skills are only offered at two German medical faculties – Augsburg and Witten-Herdecke – as part of the compulsory core curriculum. There often are facultative, university-wide courses. However, according to an informal, non-representative survey via an active mailing list of the German Medical Students’ Assocation (bvmd), these are rarely used by medical students (own survey, unpublished), presumably due to the high time and self-organizational effort involved as well as a low level of demand-oriented adaptation.

Heidelberg University also offers a course that is open to all students, but it is hardly accepted by students of human medicine due to its overly general focus.

### 2.2. Target needs assessment

In 2018, we conducted an online survey among students at the Mannheim Faculty of Medicine in order to assess the needs and expectations of medical students. 221 students took part in the survey. The majority of students were in their first (40%) and third semester (28%). 

**Figure 2:**
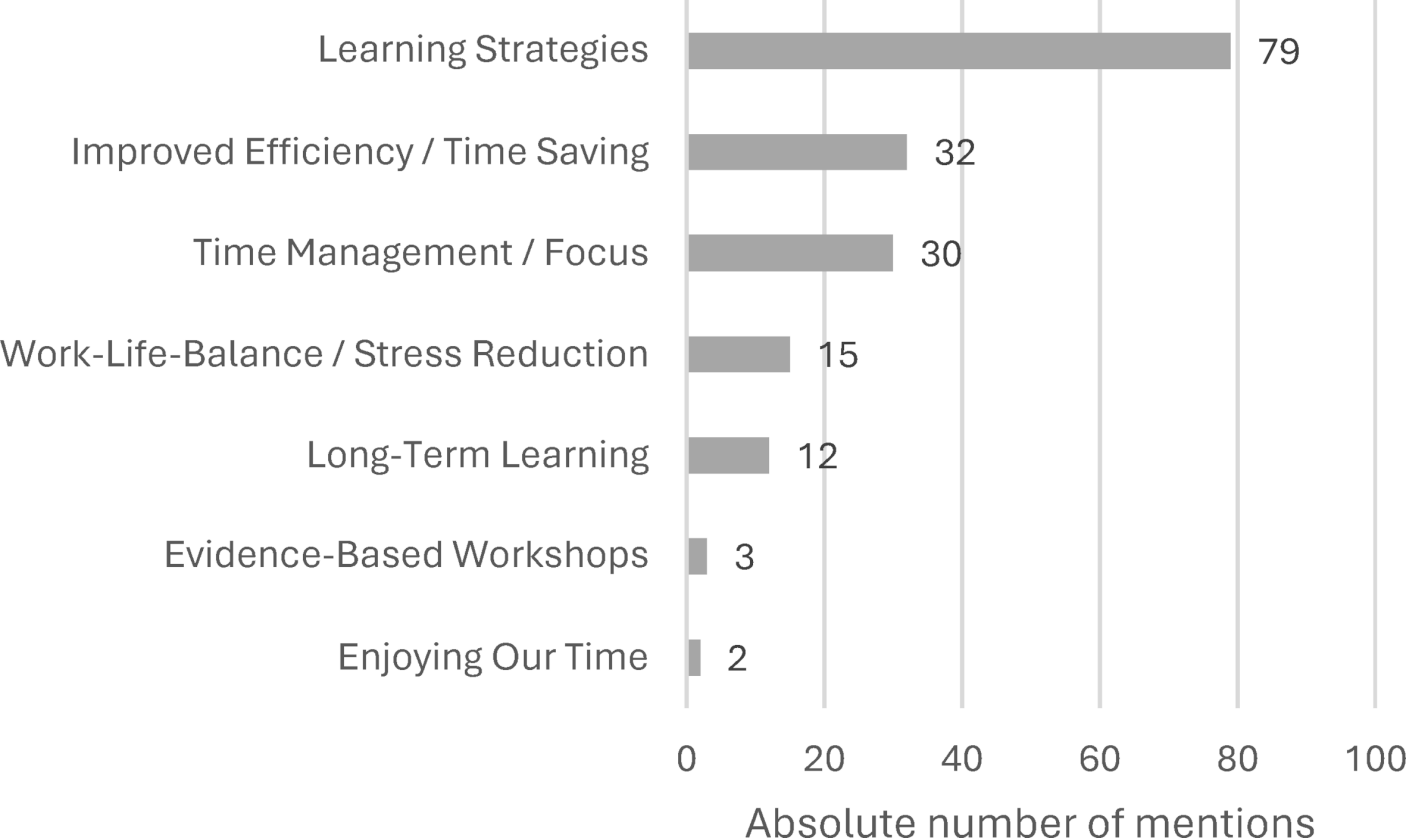
Medical students’ expectations about a student-led workshop for learning techniques

The evaluation of expectations revealed that the most frequent statement was the acquisition of learning strategies. The statements “evidence-based” and “long-term learning” are noteworthy (see figure 2 (Fig. 2)), as these were not derived from the content of the survey.

From this, we developed the concept for a peer workshop series called “study smart”.

In the initial development phase, we defined the target group as medical students at the Mannheim Medical Faculty, with a particular focus on students in their first two years of study, with the conscious aim of making the project both nationally scalable and transferable to higher semesters. 

### 2.3. Goal and objectives

We defined the following objectives based on the needs analysis:

The overarching aim of the workshop series was to support students in acquiring learning and relaxation strategies. Students should also learn to assess their own learning strategies and identify ineffective but frequently used strategies. In addition, the workshop program should help to promote self-efficacy. 

We therefore defined the following detailed learning objectives:

After active participation in the workshop program as well as preparation and follow-up, students can ...

- ... evaluate different learning strategies on their effectiveness.
- ... explain SRL and name ways of integrating SRL into everyday learning.
- ... name criteria for good goal setting and formulate their own goals accordingly.
- ... describe and implement helpful relaxation techniques.

### 2.4. Educational methods

**Figure 3:**
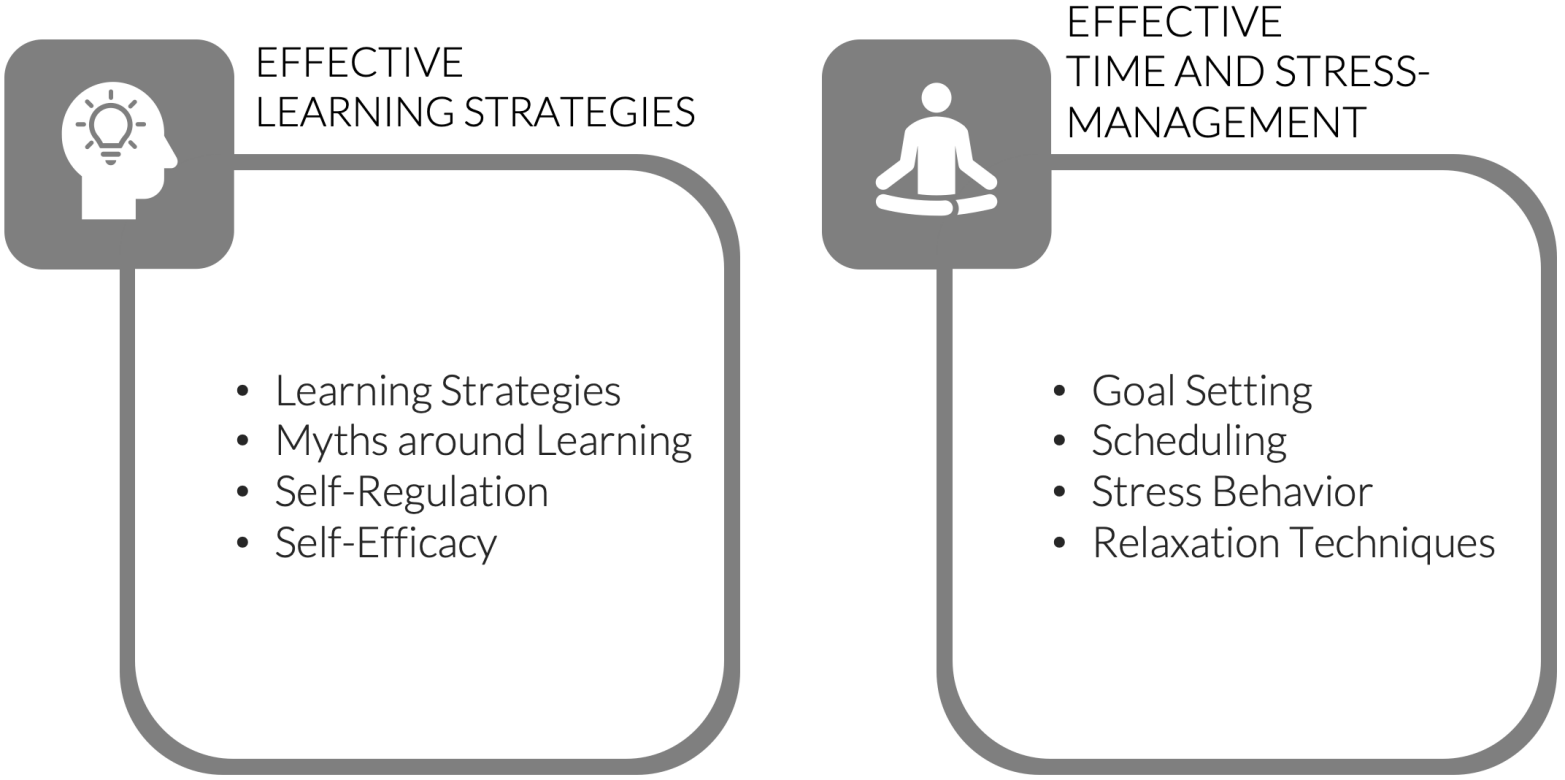
Content of the two workshops

Based on the needs analysis and the defined learning objectives, we decided to split the workshop program into two parts, focusing on effective learning techniques and self-regulated learning on the first workshop day and on time and stress management on the second (see figure 3 (Fig. 3)). 

We chose the peer-to-peer format to implement the workshop concept in order to make it easier for participants to accept the content in line with the concept of cognitive and social congruence [14].

With regard to the methods described in detail below, we built on the learning cycle described by Kolb, which divides learning into four steps [15]:

1. concrete experience
2. reflective observation
3. abstract conceptualization
4. active experimentation

**Figure 4:**
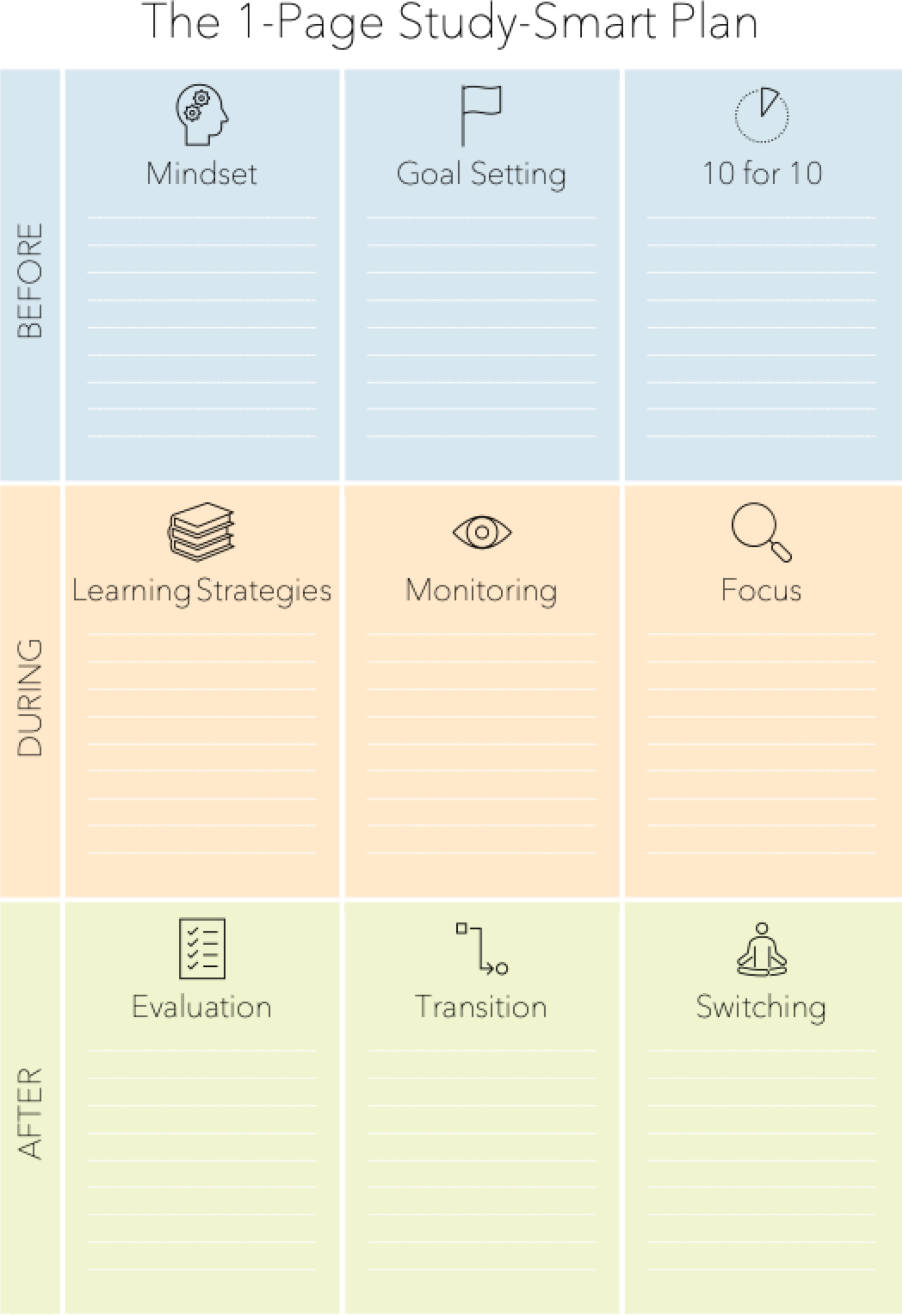
The study-smart-plan for planning individual learning behavior

In the introduction, concrete situations from the students– own everyday life are presented (step 1), which are then reflected upon (step 2) and placed in the context of learning theory concepts (step 3). The participating students then actively try out these concepts in self-experiences or individual and group work. With the help of an action plan (see figure 4 (Fig. 4)), the students prepare how they can actively try out the various concepts in the following weeks (step 4). The aim of the action plan is to make it easier for students to put what they have learned into practice in their everyday lives.

The book “Make it stick - the science of successful learning” by Brown, Roediger III and Mc Daniel served as the conceptual framework for the “effective learning techniques” part of the workshop [16]. First, frequently used learning strategies are presented and criteria for assessing them are developed, focusing on self-awareness regarding the (in-)effectiveness of the strategies used and concrete possibilities for implementation. Subsequently, the various learning myths such as the division into different “learning types” are addressed and the concepts of “self-regulation” and “self-efficacy” are developed with examples of their application [17], [16]. 

In the “time and stress management” part of the workshop, the focus is put on strategies for setting goals, promoting concentration and the principles of stress and stress reduction.

To achieve this, characteristics and effects of good goal setting are developed and concrete goals are formulated within the framework of medical studies [18]. After a brief overview of the epidemiology of mental health problems in higher education with the intention of destigmatization, the focus is on the physiological basis of the human stress response as well as evidence-based techniques for stress reduction [19], [20], [21].

### 2.5. Implementation

The workshop series was offered for the first time in November 2018 as a voluntary evening event. Originally planned as a small group format, the workshop concept had to be adjusted as over 240 students signed up for it.

Based on the feedback from the pilot workshop, we refocused the workshop series on the first year of study and contacted faculty leadership to include the workshops as an optional offer in the first semester timetable. The faculty also saw the need for such an event and so the optional integration into the timetable in the first year of study was implemented from 2019 and content was shared via internal channels (Moodle). 

Since 2020, there has been a collaboration with the Department of Medical Education Research at the Mannheim Medical Faculty to evaluate and further refine the workshop series. 

In a presentation at the German Medical Students’ Association (bvmd), it became clear that there is a national need to provide students with knowledge and skills on SRL and mental health. Study Smart thus became a bvmd project in July 2021. To scale the program nationally, we developed a concept for training peer teachers. To achieve this, we created a manual and a peer-training weekend, which has been held annually since October 2021 and has so far been attended by 25 students. 

In the winter semester 2021/22, the workshop series was launched at other faculties, including Göttingen, Rostock, Augsburg, Würzburg, Erlangen, Heidelberg and Halle, as well as several times as an online offer across faculties. Initial feedback has been very positive, indicating the implementation by trained peer trainers has worked well.

### 2.6. Evaluation and feedback

Students can evaluate their own learning success with the help of a multiple-choice exam created for this purpose, in which students can check their understanding of criteria for good goal setting or possible examples of the application of self-regulated learning in 15 questions.

By including the workshop program in the optional curriculum of the Medical Faculty Mannheim, the workshop series is evaluated via EvaSys (evasys GmbH, Lüneburg). The evaluations and verbal feedback received are used annually to further develop the workshop design.

In February 2021, a survey of participants in the workshop series and non-participants from the first year of study was conducted two months after the workshop series was held as part of the semester evaluation and served to check whether the project objective had been achieved - whether it was possible to develop a low-threshold peer teaching workshop series on evidence-based learning strategies, self-management, time management and stress management that could be transferred to faculties at other universities. The survey was conducted online using closed questions (e.g. satisfaction with own learning strategies, time and stress management on a Likert scale) and open questions (e.g. “How do you learn most effectively?”). The answers were categorized and summarized. A total of 68 students who participated in the workshops and 72 students who did not participate in the workshops and served as a control group took part in the survey.

Both groups were asked about reasons for participation or non-participation. In the first part of the survey, we compared satisfaction with learning strategies, study performance and time and stress management between workshop participants and non-participants two months after the workshops. Participants were also asked the open question: “How do you learn most effectively?” Here, we evaluated specific learning strategies that we had taught and that had previously been evaluated as efficient learning techniques according to the literature, such as “Active Recall” and the flashcard software “anki”.

In 2021, we also conducted non-structured, oral interviews with individual workshop participants. In the weeks following their participation in the workshop, we asked the participants about the most important content for them, suggestions for improvement and changes they had noticed in their own learning behavior. 

Based on the evaluation results, we continually improved the workshop series each year. For example, the dates were changed from module 1 to module 3 in the first year of study, where more students were able to take part in the workshops due to a smaller number of lectures and seminars. We also developed the 1-page plan (see figure 4 (Fig. 4)) in response to the students' request for a structured plan for the practical implementation of the workshop content.

## 3. Results

Reasons for participation were general interest in the topics (19 responses), interest in learning strategies (21 responses), interest in stress management (12 responses) and interest in better time management (1 response).

There were 39 responses regarding the reasons for non-participation. The most common reasons given were lack of time and scheduling conflicts (26 responses). The second most common reason was satisfaction with their own skills (7 responses).

The data show no significant differences in satisfaction in relation to the skills examined (see table 1 (Tab. 1)).

Here, the use of “active recall” and the flashcard software “anki” stood out with 47 and 18 mentions respectively. There was no significant difference in the use of “active recall” between workshop participants and non-participants (n=85; p=0.06). The use of anki showed a significant difference between the two groups (n=85; p=0.009), with workshop participants stating more frequently that they learned most effectively with anki (see table 2 (Tab. 2)). 

The interviews served to gain deeper insights into the individual experiences of the participants with regard to the workshops and their setup. 

During the interviews, it was found that there was a lot of exchange among the students after the workshops. For example, summaries were shared to make the knowledge imparted available to non-participants in short form. Furthermore, Anki decks were created with index cards adapted to the content of the Mannheim Medical Faculty, with groups of students continually developing them.

In total, over 1000 medical students participated in the workshops over the past five years. In addition, the “study smart” project won second place in the DACH Association for Medical Education (GMA) 2023 project prize.

## 4. Discussion

The aim of the project was to survey the needs of students and subsequently develop a series of workshops that teach students the content and possible applications of key learning skills, which is accessible and transferable to other faculties. 

There was a high level of interest among students both at the Medical Faculty Mannheim and at other faculties. The workshop series is attended by many students and has established itself as a low-threshold and frequently used offer at the Medical Faculty Mannheim. 

There was no significant difference between the control group and the intervention group in the participants’ assessment of their own satisfaction with their learning strategies, time and stress management. One cause could be distortion effects in the self-assessment (Dunning-Kruger effect), which leads to a poorer self-assessment among students who have dealt with a topic to some extent than students who have not dealt with it at all [22]. Participating students may have objectively improved their key learning skills but may have assessed themselves more critically. Another reason could be insufficient randomization of the students. This is made unlikely by the feedback from the control group: the main reason for non-participation was that the students did not have time. It is also possible that the reduction of content to two 90-minute workshops makes it more difficult to change global learning behavior. The seven-week “learning to learn” workshops at Witten/Herdecke University, for example, consist of an 8-hour day course and six 90-minute workshops [23]. These enable a more profound change in learning behavior but can only be integrated into existing curricula at great expense.

The use of active recall did not show any significant difference between the groups. One possible reason could be that this technique is now also known outside of our workshops, possibly through word-of-mouth or the sharing of summaries. Anki, on the other hand, showed a significant difference. Anki combines various concepts such as distributed learning and active recall and is therefore recommended in our workshops as a particularly effective learning technique. Our workshops may have made students aware of the importance and value of anki in their learning process, leading to greater use.

The workshops were offered at various faculties in Germany. A handbook and a peer-training weekend were developed for this purpose and held yearly. So far, 25 students have been trained. Nevertheless, there is the challenge of implementing the workshops in the long term, as the students often only offer the workshops at their own faculty for a short period of time and a continuation of the workshops is not always guaranteed. We are therefore currently developing concepts for the structured continuation of the workshops at the respective faculties. It is important to constantly inspire and train new students for the peer training. In addition, peer teachers should be encouraged to continue running the workshops or involving new students as part of a network.

When transferring the workshops to other (medical) faculties, it should be noted that the workshops must be adapted to the specific conditions at each location. In particular, the modular structure of the preclinical program at the Medical Faculty Mannheim results in location-specific priorities and challenges. We assume that a well-adapted series of workshops will also be feasible and well received at other locations. Current results from other faculties suggest that study smart can be established at other faculties as well as a low-threshold workshop series that is popular with students.

Several optional Study Smart add-ons are currently being developed, such as an “M1 special” with specific examples of how to apply the workshop content in preparation for the first state examination and an “introduction to anki” workshop.

There is still potential for longitudinal networking of the content and detailed adaptation of the workshop content.

## 5. Conclusion

It was shown that it is possible to introduce student-led workshops in a peer-teaching format on evidence-based learning techniques, time and stress management at German faculties. 

There is a continuously high demand for the Workshops by students at the Medical Faculty Mannheim and they are accepted with a high level of satisfaction. The workshop series is currently being established at several German medical faculties. A statistically significant change in student satisfaction with their learning skills could not be recorded in our survey. Regarding changes in learning behavior, there was primarily an increase in the use of the Anki flashcard software.

The workshop series was rated as particularly useful for first-year students. To enable more students to participate, each workshop date is offered on three different days. 

The long-term implementation of the workshops despite the high fluctuation of peer teachers and the longitudinal anchoring of the content in the degree course remains a challenge. Long-term implementation of the workshops at various faculties requires intensive cooperation between local groups and national project coordination.

## Authors’ ORCIDs

- Georg Gross: [0000-0001-9144-1247]
- Leon Feron: [0009-0006-3639-0700]
- Felix Beetz: [0009-0009-2857-4093]
- Nicolas Krapp: [0009-0004-8941-3836]
- Katrin Schüttpelz-Brauns: [0000-0001-9004-0724]

## Acknowledgements

We would like to thank Anna Riedel for accompanying the first implementation. We would also like to thank Ana-Maria Bordes for carrying out the evaluation and the entire Office of Student Affairs and Student Secretariat of the Medical Faculty Mannheim for their support in carrying out the workshop series. We would also like to thank Professor Bohus from the Central Institute of Mental Health in Mannheim for his advice on content and methodology. We would also like to thank all the students active in the bvmd, especially the National Officers on Medical Education.

## Competing interests

The authors declare that they have no competing interests. 

## Figures and Tables

**Table 1:**
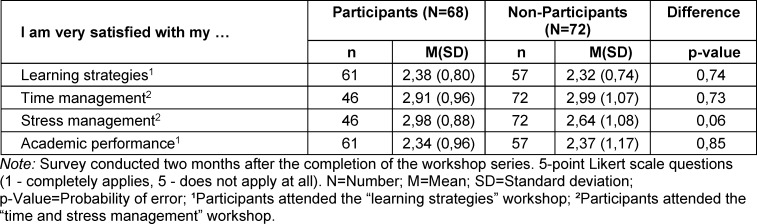
Satisfaction with learning strategies, academic performance, and time and stress management comparison between students who attended the study smart workshop and students who did not attend

**Table 2:**
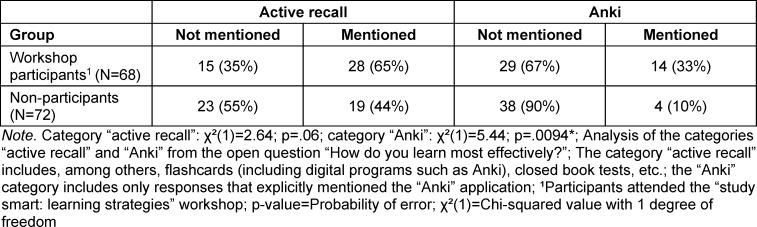
Self-assessment of the most effective personal learning strategy among medical students
